# The role of epigenetic age acceleration and social disadvantage in cardiometabolic health in southeast Louisiana women

**DOI:** 10.1186/s13148-026-02183-0

**Published:** 2026-07-02

**Authors:** Alicia K. Smith, Seyma Katrinli, Julian L. Moran, Kristyne D. Mansilla Dubon, Sierra N. Garth, Michael Simmond, Dawayland O. Cobb, Meghan Brashear, Edward J. Trapido, Erika J. Wolf, Ariane L. Rung, Nicole R. Nugent, Edward S. Peters

**Affiliations:** 1 Department of Gynecology and Obstetrics, School of Medicine, Emory University, Atlanta, USA; 2 Department of Psychiatry & Behavioral Sciences, School of Medicine, Emory University, Atlanta, USA; 3 Department of Human Genetics, School of Medicine, Emory University, Atlanta, USA; 4 School of Medicine, Emory University, Atlanta, USA; 5 Epidemiology Department, University of Nebraska Medical Center College of Public Health, Omaha, USA; 6 Epidemiology Program, Louisiana State University School of Public Health, New Orleans, USA; 7 Department of Psychiatry, National Center for PTSD at VA Boston Healthcare System, Boston University Chobanian & Avedisian School of Medicine, Boston, USA; 8 Department of Psychiatry and Human Behavior, Alpert Brown Medical School, Providence, USA; 9 Department of Pediatrics, Alpert Medical School of Brown University Box G-BH, Providence, RI 02912 USA; 10 Department of Emergency Medicine, Alpert Brown Medical School, Providence, USA

**Keywords:** Epigenetic age acceleration, DNA methylation, Cardiometabolic disease, Cardiovascular disease, Diabetes, Social disadvantage, Socioeconomic status, Area Deprivation Index, Women’s health

## Abstract

**Background:**

Cardiometabolic risk factors can disrupt DNA methylation (DNAm) patterns, accelerate cellular aging, and contribute to age-related disorders, including cardiovascular disease (CVD) and diabetes. Social disadvantage may exacerbate these biological processes, yet its relationship with epigenetic aging and cardiometabolic disease across time remains incompletely understood. We examined associations between epigenetic age acceleration and both social disadvantage and cardiometabolic diseases. We assessed whether these associations differed in each study wave.

**Results:**

Participants were drawn from The Women and Their Children’s Health (WaTCH) cohort and assessed at Wave 1 (2012–2014; *N* = 865) and Wave 3 (2023–2025; *N* = 348). At Wave 1, participants had a mean age of 46.9 ± 11.9 years and identified as Black (38.0%) or White (56.2%). Social disadvantage was assessed using an individual-level socioeconomic status (SES) index (education, income, and health insurance) and the Area Deprivation Index (ADI). DNAm was measured from blood samples to derive epigenetic age acceleration metrics (GrimAge2, PhenoAge, and DunedinPACE). Lower SES index and higher ADI were associated with greater epigenetic age acceleration and a faster pace of aging. CVD (45.2%) and diabetes (15.6%) at Wave 1 were more prevalent among participants with lower SES index, higher ADI, Black race, and higher body mass index (BMI). After adjustment for BMI, alcohol use, ADI, SES, smoking, and cell composition, both CVD and diabetes were associated with higher GrimAge2 and PhenoAge acceleration and a faster pace of aging at Wave 1. At Wave 3, diabetes was associated with all measures of epigenetic age acceleration after covariate adjustment.

**Conclusions:**

CVD and diabetes were more common among socially disadvantaged women and were associated with accelerated epigenetic aging. These findings highlight the biological correlates of social disadvantage and underscore the need for targeted public health interventions addressing social drivers of health to mitigate cardiometabolic disease risk.

**Supplementary Information:**

The online version contains supplementary material available at https://doi.org/10.1186/s13148-026-02183-0.

## Introduction

Cardiometabolic diseases, including both cardiovascular disease (CVD) and diabetes, are major public health concerns, and their incidence in women increases substantially with age. CVD remains the leading cause of mortality among women in the United States, with recent data suggesting rising rates for middle-aged women [1, 2]. Similarly, diabetes is a significant health concern among women, particularly as they age, because women with diabetes experience higher rates of adverse outcomes than men [3–5]. Modifiable behavioral factors, such as obesity and alcohol use, play a significant role in CVD and diabetes [1, 6]. In addition to individual behaviors, increasing attention has been paid to the role of the social and built environment in shaping cardiometabolic health. A growing body of literature links socioeconomic status (SES) and neighborhood-level factors, including food availability, walkability, and crime, to increased risk of CVD and diabetes [7]. Recognizing the importance of these factors for cardiometabolic health, the American Heart Association and the American Diabetes Association have issued statements calling for multilevel approaches that account for social drivers of health (SDoH), such as economic stability and neighborhood safety, in disease prevention and management strategies [8, 9]. 

DNA methylation (DNAm) regulates gene expression in response to internal and external environmental conditions [10–12] and is a mechanism whereby individual-level and neighborhood-level environments can influence cardiometabolic health. Indeed, cardiometabolic risk factors can disrupt DNAm patterns, accelerating cellular aging and providing insight into the development of age-related disorders, such as CVD and diabetes [13, 14]. Recent studies employing epigenetic clocks (DNAm-based biomarkers of biological aging) [15–17] show accelerated epigenetic aging associated with CVD and diabetes [18–25]. Still, there is a paucity of studies that have integrated epigenetic aging, SDoH, and cardiometabolic health in a multilevel analytic framework. However, studies do support the influence of SDoH on epigenetic aging. For example, studies demonstrate that both childhood and adult SES, which show distinct epigenetic patterns, associate with epigenetic age acceleration and the pace of aging [26, 27]. A recent study demonstrated that age acceleration (e.g., GrimAge) and the pace of aging mediated the association between SES and kidney function decline over 10 years, highlighting the link between SDoH, epigenetic age acceleration, and age-related chronic conditions [27]. 

In this study, we leverage phenotypic data and biospecimens from the Women and Their Children’s Health (WaTCH) study, a longitudinal cohort established in response to the Deepwater Horizon Oil Spill [28]. The cohort includes women residing in southeastern Louisiana, a region characterized by high rates of chronic disease, socioeconomic disadvantage, and persistent racial and geographic health disparities [29]. Louisiana consistently ranks among the worst states for CVD and diabetes outcomes, and life expectancy among women—particularly Black women—lags well behind national averages [29, 30]. The goal of this study is to describe the cross-sectional associations between age acceleration and both social disadvantage and cardiometabolic disorders in women from the WaTCH cohort. To accomplish this, we calculated three epigenetic clocks: (1) GrimAge2 [31], an updated version of GrimAge [32] that uses DNAm-based surrogates to capture indicators of morbidity and all-cause mortality risk, including hemoglobin A1C and CRP estimates; (2) PhenoAge [33], which incorporates DNAm-based indicators of clinical biomarkers of organ systems, such as serum glucose levels; and (3) DunedinPACE, which estimates the pace of biological aging related to longitudinal within-person changes across organ systems [34]. We (1) test associations between each epigenetic clock and indices of social disadvantage; (2) evaluate whether those with and without cardiometabolic disease (e.g., CVD and diabetes) differ by social disadvantage; (3) evaluate whether those with and without cardiometabolic disease (e.g., CVD and diabetes) differ by age acceleration independent of social and clinical covariates; and (4) because risk of cardiometabolic disorders increases with age, re-examine these associations in a subset of the cohort that participated in a subsequent wave of the study conducted ~ 10 years later. We hypothesize that higher age acceleration would be associated with an increased risk of CVD and diabetes, even after adjusting for social disadvantage, and that this risk will persist over time. This work contributes to our understanding of how social and biological factors may jointly reflect health trajectories in vulnerable populations.

## Methods

### Study design and population

Data were drawn from the Women and Their Children’s Health (WaTCH) Study, a longitudinal cohort established to investigate physical and mental health outcomes among women affected by the 2010 Deepwater Horizon Oil Spill [35]. Detailed study design and recruitment procedures have been described previously [35]. Briefly, women residing in seven parishes in southeastern Louisiana were recruited between 2012 and 2014 using an address-based sampling frame and completed a baseline telephone interview (Wave 1), with an option for blood collection. Follow-up interviews were conducted during Wave 2 (2014–2016). A third wave of data collection (Wave 3) occurred between January 24, 2023, and September 30, 2025. For Wave 3, participants’ vital status, current address, and phone number were confirmed using LexisNexis SmartLinx. Introductory letters for Wave 3 described the study, requested another blood sample, and invited survey participation. Wave 3 participants were invited by trained interviewers from the University of Nebraska Bureau of Sociological Research (BOSR). Willing participants received a study packet containing survey response cards, consent form, and blood collection kit with a pre-paid return shipping label. A telephone interview with the BOSR team and an in-home phlebotomist appointment (provided by ExamOne) were scheduled. Following collection, blood samples were shipped overnight for laboratory processing. Women were contacted up to 12 times by telephone, and a text message was sent to those with cellphones. Participants who preferred to complete the survey themselves could request an email link to the online version. All study data were collected and managed using REDCap [36]. Per eFigure 1 in Additional File 1, this study focused on the women who provided a Wave 1 blood sample and participated in the Wave 2 interview (*N* = 865) and the subset of the cohort provided a blood sample in Wave 3 (*N* = 348). Analyses were conducted cross-sectionally at each wave using data from Waves 1 and 3.

All participants provided written informed consent. The study was approved by the Institutional Review Board of Louisiana State University Health Sciences Center–New Orleans and was conducted in accordance with the Declaration of Helsinki. This report follows the STROBE guidelines for observational studies.

### Sociodemographic and clinical measures

Sociodemographic characteristics, including age, race, education, employment status, household income, and health insurance status, were obtained by self-report during structured interviews. Race response options were consistent with standardized surveys at the time and included: Caucasian (White), African-American (Black), Asian/Pacific Islander, American Indian or Native American, Other, Don’t Know, or Refused. Those who reported Asian/Pacific Islander, more than one race, didn’t know or refused were included in the Other category. Consistent with our prior work [37], the category of high school graduate included those with a GED, vocational training, community college, or some college. Income was coded as < $20,000, $20,001-$50,000, $50,001-$80,000, and > $80,000. Health insurance was coded as private insurance, public insurance (e.g. Medicare or Medicaid), and no insurance.

Alcohol use was based on self-report and categorized as never, former or current use. Body mass index (BMI) was assessed at the home visit (Wave 1) or via self-report (Wave 3).

### Socioeconomic status and neighborhood deprivation

To capture individual-level socioeconomic position, we constructed a composite socioeconomic status (SES) index using education, household income, and health insurance status. Missing values for the contributing variables were imputed with the *knn* function in the VIM package (v6.2.2). Age was included as an auxiliary variable in the K-nearest neighbor (KNN) imputation to help improve the accuracy but was not included in the subsequent principal components analysis (PCA). A PCA was conducted on data from Waves 1 and 3 using the *princomp* function. The first PC (Eigenvalue = 1.76) accounted for 59% of the variance and was used as our SES index.

Residential addresses at each Wave were geocoded with ArcGIS Pro version 3.2 (Esri, Redlands, CA) to assign a corresponding census block group. Participants were assigned both Louisiana-specific and national 2012 Area Deprivation Index (ADI) ranks at the block group level, derived from the Neighborhood Atlas. The ADI is a validated composite score based on 17 census measures from the 5-year American Community Survey. Higher ADI scores indicate neighborhoods with greater disadvantage [38, 39]. Because some participants moved out of state between Waves, this study uses national ADI.

### DNA methylation profiling and epigenetic aging measures

DNA was isolated from blood using the QIAamp DNA Mini Kit (Qiagen), and DNAm was interrogated using the MethylationEPICv2 BeadChip (Illumina). Prior to calculating age acceleration, low quality probes with detection p-values > 0.05 were set as missing. Probes with missing data for greater than 5% of samples and samples with missing data for greater than 5% of probes were removed. Data was background and dye-bias corrected, quantile normalized, and adjusted for probe-type bias using ENmix [40]. The probes flagged as inaccurately mapped or poorly performing were removed [41]. Replicate probes were handled according to the recommendations of Peters and colleagues [41]. Cell composition (i.e., the proportion of CD8T, CD4T, natural killer (NK), B cells, monocytes, and neutrophils) was imputed using the Robust Partial Correlation (RPC) method in *Epidish* [42] using reference data [43]. 

DNAm age was calculated using methods described by Lu (GrimAge2) [31] and Levine (PhenoAge) [33]. PhenoAge calculations used the PC-based approach, which demonstrates strong intra-class correlations (ICCs) between technical replicates compared to traditionally-calculated CpG clocks [44]. We then performed an ANOVA to examine whether there was any difference in epigenetic age acceleration for each clock based on plate, chip or position and found no evidence of residual confounding by technical factors. Epigenetic age acceleration was calculated as the residual between DNAm age and chronological age. The pace of aging was estimated using the DunedinPACE clock as described by Belsky [34]. The pace of aging estimate provided is scaled to a mean of 1 year of biological aging per year of chronological aging to facilitate interpretation.

### Cardiometabolic outcomes

At Waves 1 and 3, participants were asked if they had been diagnosed with heart disease, high blood pressure, a heart attack, angina, congestive heart failure, or a stroke in a structured survey interview. These variables were collectively categorized as having CVD. Participants were also asked if they had been diagnosed with diabetes.

### Statistical analysis

Descriptive statistics were calculated for sociodemographic and clinical variables. Means were compared between groups using t-tests, and percentages were compared using χ^2^ tests. Unadjusted linear regressions were used to test the bivariate association between social disadvantage (SES Index and ADI) and age acceleration at each Wave. Differences in sociodemographic and clinical variables between individuals with and without CVD and diabetes were compared using logistic regression. For categorial variables, the largest group served as the reference category.

To test the bivariate association between cardiometabolic disorders and age acceleration, we used unadjusted logistic regression at each respective wave (model 1). We identified confounding variables through a Directed Acyclic Graph (eFigure 2 in Additional File 1) using Dagitty.net [45]. The minimally sufficient adjustment set (MSAS) identified for estimating the direct effect of age acceleration on cardiometabolic disorders was BMI, alcohol use, ADI, SES, and smoking. Multivariable logistic regression models then evaluated the association between CVD or diabetes and age acceleration controlling for these covariates and cell composition (model 2). To aid interpretation, standardized odds ratios were calculated by scaling the focal predictor prior to model fitting, such that the odds ratios reflect the change in odds associated with a 1 SD increase in the predictor. For linear models, standardized β coefficients were calculated by scaling both the focal predictor and the outcome prior to model fitting, such that coefficients reflect the change in the outcome per 1 SD increase in the predictor. Our primary analysis was conducted in data from Wave 1 and then repeated in the subset that participated in Wave 3.

All hypotheses were considered with 2-sided tests, assuming a significance threshold of *p* < 0.05. Analyses were conducted in R version 4.5.

## Results

### WaTCH cohort demographic & characteristics

At Wave 1, 865 women from the WaTCH Study (mean [SD] age, 46.9 [11.9] years) were included in the analysis (Table 1), self-reporting as Black (38.0%), Native American (2.2%), White (56.2%), or Other (3.5%) racial groups, with an average BMI of 32.5. CVD was present in 45.2% of participants while diabetes was present in 15.6%.

**Table 1:** Characteristics of the WATCH participants (*N* = 865) in this study

Characteristic	Wave 1 (*N* = 865) 8/2012-6/2014	Wave 3 (*N* = 348) 1/2023-9/2025	*p*-value
Age, years (SD)	46.9 (11.9)	56.9 (11.1)	< 0.001
Race, No. (%)			0.15
Black	329 (38.0%)	132 (37.9%)	
Native American	19 (2.2%)	8 (2.3%)	
White	486 (56.2%)	204 (58.6%)	
Other	30 (3.5%)	4 (1.1%)	
Employment, No. (%)			< 0.001
Unemployed	310 (35.8%)	19 (5.5%)	
Retired	88 (10.2%)	69 (19.8%)	
Part-time	12 (1.4%)	71 (20.4%)	
Other	103 (11.9%)	38 (10.9%)	
Full-time	351 (40.6%)	151 (43.4%)	
SES Index, unit (SD)	-0.15 (1.33)	-0.07 (1.47)	0.35
ADI, unit (SD)	63.3 (19.6)	69.0 (18.7)	< 0.001
Lifetime Smoking History, No. (%)	314 (36.3%)	102 (29.3%)	< 0.001
Alcohol Use, No. (%)			< 0.001
Never	393 (45.4%)	106 (30.5%)	
Former	230 (26.6%)	60 (17.2%)	
Current	238 (27.5%)	180 (51.7%)	
BMI, kg/m^2^ (SD)	32.5 (9.12)	32.5 (8.55)	0.99
CVD, No. (%)	391 (45.2%)	133 (38.2%)	0.04
Diabetes, No. (%)	135 (15.6%)	67 (19.3%)	0.13

Race was missing for 1 observation. Employment was missing for 1 participant at Wave 1. 28 participants reported not knowing or refusing to provide their annual household income at Wave 1 and 8 at Wave 3. 2 participants did not report insurance and 3 did not report BMI at Wave 3. ADI was missing for 2 participants at Waves 1 and 3. 1 participant was missing data on CVD at Wave 1 and Wave 3. 1 participant was missing data on diabetes at Waves 1 and 3

*SES*  Socioeconomic status, *ADI*  Area deprivation index, *BMI*  Body mass index

### Disadvantage associates with age acceleration

In a prior study [46], we reported Wave 1 GrimAge acceleration and a faster pace of aging in participants who report Black or Native American race relative to those who report White race. In this study, we examined SES index and ADI as indicators of economic and neighborhood disadvantage at Waves 1 and 3 (eTable 1 in Additional File 1). Lower SES is associated with higher GrimAge2 acceleration, PhenoAge acceleration, and a faster pace of aging. Higher ADI is associated with higher GrimAge acceleration and a faster pace of aging. These results are consistent even when controlling for age, smoking, BMI, and cell composition (eTable 2 in Additional File 1), with PhenoAge acceleration also associating with ADI at Wave 1 in the adjusted models.

### CVD and diabetes are more common in disadvantaged groups

We noted differences in some characteristics for participants who reported a diagnosis of either CVD or diabetes (Table 2) at Wave 1. Compared to participants without CVD, those with CVD report lower overall SES and residing in more disadvantaged neighborhoods. Participants with CVD were also older, more likely to report Black race, had higher average BMIs, were less likely to currently drink alcohol, and were more likely to be unemployed or retired. Compared to participants without diabetes, those with diabetes also reported lower SES and living in more disadvantaged neighborhoods. They were older, more likely to report Black race, had higher average BMIs, were more likely to be unemployed or retired, and were more likely to report a history of smoking.

**Table 2:** Sociodemographic differences associated with CVD and diabetes at Wave 1

Characteristic	CVD OR (95% CI)	*p*-value	Diabetes OR (95% CI)	*p*-value
Age	1.08 (1.07 to 1.10)	< 0.001	1.04 (1.03 to 1.06)	< 0.001
Race				
Black	2.16 (1.62 to 2.88)	< 0.001	2.16 (1.48 to 3.19)	< 0.001
Native American	1.51 (0.59 to 3.82)	0.38	1.96 (0.70 to 4.72)	0.55
White	[reference]	NA	[reference]	NA
Other	1.68 (0.80 to 3.54)	0.17	1.96 (0.70 to 4.72)	0.16
Employment				
Unemployed	2.15 (1.57 to 2.95)	< 0.001	2.07 (1.34 to 3.22)	0.001
Retired	6.46 (3.81 to 11.39)	< 0.001	2.42 (1.31 to 4.39)	0.004
Part-time	0.95 (0.25 to 3.08)	0.93	1.65 (0.25 to 6.55)	0.53
Other	0.94 (0.58 to 1.48)	0.78	1.19 (0.59 to 2.28)	0.61
Full-time	[reference]	NA	[reference]	NA
SES Index	0.68 (0.61 to 0.75)	< 0.001	0.74 (0.64 to 0.85)	< 0.001
ADI	1.02 (1.01 to 1.02)	< 0.001	1.02 (1.01 to 1.03)	< 0.001
Lifetime Smoking History	1.22 (0.92 to 1.61)	0.16	1.55 (1.07 to 2.25)	0.021
Alcohol Use				
Never	[reference]	NA	[reference]	NA
Former	0.86 (0.62 to 1.19)	0.35	1.57 (1.03 to 2.38)	0.035
Current	0.48 (0.34 to 0.67)	< 0.001	0.69 (0.42 to 1.12)	0.14
BMI	1.05 (1.03 to 1.07)	< 0.001	1.05 (1.03 to 1.07)	< 0.001

*SES*  Socioeconomic status, *ADI*  Area deprivation index, *BMI*  Body mass index

*P*-values are computed from bivariate logistic regressions comparing the disease group to those without CVD or diabetes

### CVD and diabetes associate with age acceleration

In unadjusted models (Table 3, Model 1), participants with CVD had higher Wave 1 age acceleration and a faster pace of aging. In models adjusted for BMI, alcohol use, ADI, SES, smoking, and cell composition (Table 3, Model 2), CVD remained associated with GrimAge2 acceleration, PhenoAge acceleration, and a faster pace of aging. Similarly, in unadjusted models (Table 3, Model 1), participants with diabetes had higher Wave 1 age acceleration and a faster pace of aging. In fully adjusted models (Table 3, Model 2), diabetes remained associated with GrimAge2 acceleration, PhenoAge acceleration, and a faster pace of aging. Additional adjustment (beyond residualizing epigenetic age estimates) for chronological age did not alter the results (eTable 3 in Additional File 1).

**Table 3:** Multivariate association of age acceleration and both CVD and diabetes at Wave 1

	Model	GrimAge2 Acceleration		PhenoAge Acceleration		Pace of Aging	
		OR (95% CI)*	p-value	OR (95% CI)*	p-value	OR (95% CI)*	p-value
CVD	Model 1	1.40 (1.22 to 1.61)	< 0.001	1.34 (1.17 to 1.55)	< 0.001	1.63 (1.42 to 1.89)	< 0.001
	Model 2	1.21 (1.03 to 1.43)	0.02	1.22 (1.03 to 1.47)	0.03	1.36 (1.14 to 1.62)	< 0.001
Diabetes	Model 1	1.53 (1.28 to 1.83)	< 0.001	1.61 (1.34 to 1.95)	< 0.001	1.91 (1.58 to 2.32)	< 0.001
	Model 2	1.35 (1.10 to 1.67)	0.004	1.57 (1.25 to 1.98)	< 0.001	1.64 (1.31 to 2.07)	< 0.001

Model 1 was estimated with no covariates. Model 2 controls for BMI, alcohol use, ADI, SES, smoking, and cell composition. *OR (95% CI): Standardized Odds Ratio with 95% confidence intervals

### Persistence of associations in wave 3

Given the sample attrition between Waves 1 and 3, we compared the baseline data for the retained and non-retained participants (eTable 4 in Additional File 1). Compared to the baseline information for the full cohort, the non-retained subjects have no significant differences. The subset of retained subjects has slightly higher SES (*p*=0.04) but no other significant differences. We then compared the retained participants characteristics at Waves 1 and 3. Relative to Wave 1, the subset of participants with Wave 3 data reported being ~ 10 years older and differed in employment status (Table 1). Though there was no difference in SES, Wave 3 participants lived in more disadvantaged neighborhoods. They had lower lifetime history of smoking but were more likely to currently drink alcohol. They were less likely to report CVD and had consistent diabetes rates.

SES at Wave 3 associated with GrimAge2 and PhenoAge acceleration along with a faster pace of aging, with similar magnitudes and directions of effect in bivariate (eTable 1 in Additional File 1) and fully adjusted models (eTable 2 in Additional File 1). Wave 3 ADI also associated with GrimAge2 acceleration and a faster pace of aging. However, the association between ADI and PhenoAge acceleration did not retain significance in the fully adjusted Wave 3 model.

Consistent with our Wave 1 results, those with CVD at Wave 3 were older, more likely to report Black race, being retired, and were less likely to currently drink alcohol (eTable 5 in Additional File 1). Those with CVD were also more likely to report lower overall SES and reside in more disadvantaged neighborhoods. Compared to participants without diabetes, those with diabetes at Wave 3 were also older and had lower SES. There were no associations at Wave 3 between BMI and either cardiometabolic disorder.

At Wave 3, unadjusted models (Table 4, Model 1), showed that participants with CVD had higher GrimAge2 acceleration, PhenoAge age acceleration and a faster pace of aging. However, this association did not remain in models adjusted for BMI, alcohol use, ADI, SES, smoking, and cell composition (Table 4, Model 2). Examining diabetes at Wave 3, unadjusted models (Table 4, Model 1), revealed that participants with diabetes had higher GrimAge2 acceleration and a faster pace of aging, though fully adjusted models (Table 4, Model 2), showed that diabetes was associated with GrimAge2 acceleration, PhenoAge acceleration, and a faster pace of aging. Additional adjustment (beyond residualizing epigenetic age estimates) for chronological age did not alter the results (eTable 3 in Additional File 1).

**Table 4:** Multivariate association of age acceleration and both CVD and diabetes at Wave 3

	**Model**	GrimAge2 Acceleration		PhenoAge Acceleration		Pace of Aging	
		OR (95% CI)*	***p*** **-value**	OR (95% CI)*	***p*** **-value**	OR (95% CI)*	***p*** **-value**
CVD	Model 1	1.34 (1.08 to 1.67)	0.01	1.25 (1.01 to 1.56)	0.046	1.40 (1.13 to 1.75)	0.003
	Model 2	1.19 (0.90 to 1.58)	0.21	1.18 (0.86 to 1.61)	0.31	1.31 (0.98 to 1.75)	0.07
Diabetes	Model 1	1.59 (1.23 to 2.06)	< 0.001	1.22 (0.94 to 1.59)	0.14	1.57 (1.21 to 2.06)	< 0.001
	Model 2	1.81 (1.29 to 2.57)	< 0.001	1.58 (1.07 to 2.36)	0.02	1.98 (1.39 to 2.88)	< 0.001

Model 1 was estimated with no covariates. Model 2 controls for BMI, alcohol use, ADI, SES, smoking, and cell composition. Note that 3 participants missing Wave 3 BMI were excluded from this analysis

*OR (95% CI): Standardized Odds Ratio with 95% confidence intervals

## Discussion

The goal of this study was to examine the association between epigenetic age acceleration, social disadvantage, and cardiometabolic disorders in women. We leveraged three epigenetic clocks, each of which use a set of CpG sites to capture DNAm surrogates of different age-related indicators. Notably, the GrimAge2 [31] and PhenoAge [33] clocks capture cumulative exposures whereas the DunedinPACE [34] clock models prospective risk for physiological decline and morbidity. Thus, we would not necessarily expect all clocks to give similar results as they capture different aspects of the aging process.

Consistent with prior studies linking socioeconomic adversity to accelerated epigenetic aging [47–49], we observed that Wave 1 age acceleration is more pronounced in women with lower SES and who reside in more disadvantaged neighborhoods across all 3 epigenetic clocks. These findings support the idea that chronic exposure to social disadvantage may increase biological wear and tear, thereby accelerating cellular and systemic aging. Our results expand this research by showing that these disparities continue in an all-female cohort and across various epigenetic measures.

We observed that participants with CVD at Wave 1 exhibited significantly greater GrimAge2 and PhenoAge acceleration as well as a faster pace of aging. These associations persisted even after adjustment for clinical and socioeconimic covariates and remained consistent in analyses that also adjusted for chronological age. At Wave 3, epigenetic age acceleration only associated with CVD in the unadjusted models and did not survive covariate adjustment. Similar associations between GrimAge and cardiovascular outcomes have been observed by others [32, 50], reinforcing the construct that epigenetic aging captures cumulative biological insults relevant to cardiovascular health. The attenuation of these associations at Wave 3 may reflect reduced sample size and CVD prevalence, as well as potential survival bias among participants who remained in follow-up.

At Wave 1, participants with diabetes exhibited significantly greater GrimAge2 and PhenoAge age acceleration along with a faster pace of aging, even after adjusting for covariates and chronological age. At Wave 3, the fully adjusted models for all three epigenetic clocks remained associated with diabetes, with similar magnitudes and directions of effect. Our associations of epigenetic age acceleration with diabetes across both waves align with emerging evidence that accelerated epigenetic aging predicts insulin resistance, glucose dysregulation, and metabolic syndrome [19, 20]. 

Collectively, these data support future studies that evaluate whether age acceleration may serve as an intermediary mechanism linking social disadvantage to cardiometabolic disease. More broadly, the convergence across diverse studies strengthens the hypothesis that DNAm-based aging reflects shared biological pathways underlying metabolic and cardiovascular health. From a public health perspective, these results underscore the importance of addressing social determinants of health not only as predictors of behavioral and clinical risk factors, but also as drivers of biologic aging processes that may predispose women to an earlier onset of chronic disease. If validated in longitudinal and intervention studies, measures of epigenetic age acceleration could serve as sensitive biomarkers to identify populations at greatest risk and to evaluate the impact of upstream policy, neighborhood, and behavioral interventions aimed at reducing health inequities across the life course.

### Strengths and limitations

This study has strengths, including a well-characterized cohort that has been followed for over 10 years. However, some limitations should be noted. First, the study was conducted exclusively in women, so the results may not be generalizable to men. Indeed some research has observed sex differences in CVD and diabetes and certain aging clocks [51, 52]. Second, the reduced sample size at Wave 3 and differences in demographic and clinical characteristics relative to Wave 1 may limit power to detect associations at the same level observed at Wave 1, particularly if survivor bias occurred. However, inclusion of the Wave 3 data, taken over the 10-year period in which cardiometabolic disease risk increases in women does provide overall support for the associations observed at Wave 1. Third, disease status for each participant was based on self-report, which has the potential for misclassification. Fourth, given the cross-sectional nature of the study, we cannot determine if age acceleration results from living in disadvantaged conditions nor can we determine if age acceleration precedes development of CVD or diabetes. However, prior epidemiological studies of incident cases suggest that age acceleration precedes CVD and diabetes [18, 20, 21] and that epigenetic age acceleration mediated the relationship between SDoH and age-related chronic disease [27]. Future research should explore the temporal dynamics of age acceleration in relation to disease onset and progression and in context of social disadvantage. Finally, it is important to acknowledge that the rates of cardiometabolic disorders, socioeconomic disadvantage, and neighborhood disadvantage were not evenly distributed by race. Participants who identified as Black were more likely to report CVD, diabetes, lower SES, and higher neighborhood disadvantage. In our DAG, race was conceptualized as a social construct that acts upstream of socioeconomic status and neighborhood disadvantage, which, in turn, captures structural inequities that influence both epigenetic aging and cardiometabolic risk. Accordingly, the MSAS included SES, ADI, and other cardiometabolic risk factors, but not race. However, race may also reflect genetic ancestry, and many of the epigenetic clocks employed in this study were trained in European ancestry populations and may not perform comparably among diverse ancestries or diverse socioeconomic conditions. In this study, the correlation between DNAm age and chronological age was comparable when stratified by race, though future studies should be mindful of the systematic factors that link race and social disadvantage.

## Conclusions

Our findings suggest that CVD and diabetes are not only more prevalent among disadvantaged groups but are also associated with epigenetic age acceleration, providing further evidence that social and biological processes are integrally linked. These results are consistent with the “weathering hypothesis,” which proposes that cumulative effects of chronic socioeconomic disadvantage result in accelerated biological aging and higher rates of age-related health problems [53]. It also supports the idea that structural inequities, including racial, economic, and neighborhood disadvantage, play critical roles in long-term cardiometabolic health. Interventions that address SDoH, including economic inequality and neighborhood disadvantage, may ultimately help reduce biological aging and its effects on morbidity and mortality in women.

## Supplementary Information

Supplementary Material 1.

Supplementary Material 2.

Supplementary Material 3.

## Data Availability

All data analyzed during this study are included in this published article. Additional File 2 contains the data for Wave 1, and Additional File 3 includes the data for Wave 3. Code for this analysis is available at: https://github.com/skatrinli/WATCH-cardiometabolic.
