# Supplementary material for: The role of epigenetic age acceleration and social disadvantage in cardiometabolic health in southeast Louisiana women
Source: Clin Epigenetics. 2026 Jul 2;18:170. doi: 10.1186/s13148-026-02183-0 (PMC13595649; doi:10.1186/s13148-026-02183-0)
Supplement: Supplementary file 1 [file 13148_2026_2183_MOESM1_ESM.docx]

**eFigure 1. Study flow diagram.**

**eFigure 2. Directed acyclic graph (DAG) depicting the relationships between the epigenetic age acceleration (EAA) and cardiometabolic disease**. Green lines indicate causal or mediating paths. Pink lines indicate confounding paths. The minimally sufficient adjustment set (MSAS) for direct effect of EAA on cardiometabolic disease = BMI, alcohol use, ADI, SES, and tobacco use. Generated with daggity.net.

**eTable 1. Bivariate associations between economic and neighborhood disadvantage and epigenetic age acceleration at Waves 1 and 3.**

|  | **Wave** | **GrimAge2 acceleration** | | **PhenoAge acceleration** | | **Pace of Aging** | |
| --- | --- | --- | --- | --- | --- | --- | --- |
|  |  | ***β (95% CI)*** | ***p-value*** | ***β (95% CI)*** | ***p-value*** | ***β (95% CI)*** | ***p-value*** |
| **SES** | Wave 1 | -0.29 (-0.36 to -0.23) | <.001 | -0.16  (-0.23 to -0.01) | <.001 | -0.30  (-0.36 to -0.24) | <.001 |
| **ADI** | Wave 1 | 0.16  (0.10 to 0.23) | <.001 | 0.50  (-0.02 to 0.12) | .15 | 0.23  (0.17 to 0.30) | <.001 |
| **SES** | Wave 3 | -0.43  (-0.53 to -0.34) | <.001 | -0.18  (-0.28 to -0.07) | <.001 | -0.37  (-0.47 to -0.27) | <.001 |
| **ADI** | Wave 3 | 0.32  (0.22 to 0.42) | <.001 | 0.14  (0.04 to 0.25) | .007 | 0.25  (0.15 to 0.35) | <.001 |

*β (95% CI)* represents standardized regression beta values and 95% confidence intervals.

**eTable 2. Multivariate associations between economic and neighborhood disadvantage and epigenetic age acceleration at Waves 1 and 3, controlling for age, smoking, BMI, and cell composition.**

|  | **Wave** | **GrimAge2 Acceleration** | | **PhenoAge acceleration** | | **Pace of Aging** | |
| --- | --- | --- | --- | --- | --- | --- | --- |
|  |  | ***β (95% CI)*** | ***p-value*** | ***β (95% CI)*** | ***p-value*** | ***β (95% CI)*** | ***p-value*** |
| **SES** | Wave 1 | -0.29  (-0.35 to -0.23) | <.001 | -0.17  (-0.22 to -0.11) | <.001 | -0.22  (-0.28 to -0.16) | <.001 |
| **ADI** | Wave 1 | 0.15  (0.09 to 0.21) | <.001 | 0.07  (0.01 to 0.13) | .014 | 0.18  (0.12 to 0.24) | <.001 |
| **SES** | Wave 3* | -0.43  (-0.53 to -0.34) | <.001 | -0.18  (-0.26 to -0.09) | <.001 | -0.29  (-0.38 to -0.20) | <.001 |
| **ADI** | Wave 3* | 0.27  (0.18 to 0.37) | <.001 | 0.08  (-0.01 to 0.16) | .08 | 0.16  (0.07 to 0.025) | <.001 |

* Note that 3 participants missing BMI at Wave 3 were excluded from this analysis.

*β (95% CI)* represents standardized regression beta values and 95% confidence intervals.

**eTable 3. Multivariable sensitivity analysis of age acceleration and both CVD and diabetes. Models controls for** BMI, alcohol use, ADI, SES, and smoking, **cell composition, and chronologic age.**

|  | **Wave** | **GrimAge2 Acceleration** | | **PhenoAge Acceleration** | | **Pace of Aging** | |
| --- | --- | --- | --- | --- | --- | --- | --- |
|  |  | **OR (95% CI)** ^+^ | **p-value** | **OR (95% CI)** ^+^ | **p-value** | **OR (95% CI)** ^+^ | **p-value** |
| **CVD** | Wave 1 | 1.28  (1.08 to 1.52) | .006 | **1.43**  **(**1.18 to 1.75) | <.001 | **1.30**  (1.08 to 1.57) | .006 |
|  | Wave 3* | 1.24  (0.94 to 1.65) | .14 | **1.24**  **(**0.90 to 1.71) | .20 | **1.27**  (0.95 to 1.71) | .10 |
| **Diabetes** | Wave 1 | 1.42  (1.15 to 1.75) | <.001 | 1.75  (1.38 to 2.24) | <.001 | 1.62  (1.29 to 2.04) | <.001 |
|  | Wave 3* | 1.89  (1.34 to 2.70) | <.001 | 1.65  (1.12 to 2.48) | .01 | 1.94  (1.36 to 2.81) | <.001 |

* Note that 3 participants missing BMI at Wave 3 were excluded from this analysis.

^+^OR (95% CI): Standardized Odds Ratio with 95% confidence intervals.

**eTable 4: Characteristics of the baseline variables for retained and non-retained participants.**

| **Characteristic** | **Wave 1**  **(N=865)** | **Retained**  **(N=348)** | **Non-retained (N=517)** | **p-value**  **(retained)** | **p-value**  **(non-retained)** |
| --- | --- | --- | --- | --- | --- |
| Age, years (SD) | 46.9 (11.9) | 46.5 (11.0) | 47.2 (12.5) | .56 | .67 |
| Race, No. (%)  Black  Native American  White  Other | 329 (38.0%)  19 (2.2%)  486 (56.2%)  30 (3.5%) | 132 (37.9%)  8 (2.3%)  204 (58.6%)  4 (1.1%) | 197 (38.1%)  11 (2.1%)  282 (54.5%)  26 (5.0 %) | .15 | .55 |
| Employment, No. (%)  Unemployed  Retired  Part-time  Other  Full-time | 310 (35.8%)  88 (10.2%)  12 (1.4%)  103 (11.9%)  351 (40.6%) | 108 (31.0%)  33 (9.5%)  3 (0.9%)  50 (14.4%)  154 (44.3%) | 202 (39.1%)  55 (10.6%)  9 (1.7%)  53 (10.3%)  197 (38.1%) | .36 | .64 |
| SES Index, unit (SD) | -0.15 (1.33) | 0.03 (1.33) | -0.26 (1.32) | .04 | .11 |
| ADI, unit (SD) | 63.3 (19.6) | 61.61 (19.36) | 64.47 (19.73) | .17 | .30 |
| Lifetime Smoking History, No. (%) | 314 (36.3%) | 102 (29.3%) | 212 (24.5%) | .02 | .09 |
| Alcohol Use, No. (%)  Never  Former  Current | 393 (45.4%)  230 (26.6%)  238 (27.5%) | 153 (44.0%)  84 (24.1%)  110 (31.6%) | 240 (46.4%)  146 (28.2%)  128 (24.8%) | .34 | .52 |
| BMI, kg/m^2^ (SD) | 32.5 (9.12) | 32.94 (9.41) | 32.22 (8.93) | .46 | .56 |
| CVD, No. (%) | 391 (45.2%) | 145 (41.7%) | 246 (47.6%) | .30 | .43 |
| Diabetes, No. (%) | 135 (15.6%) | 58 (16.7%) | 77 (14.9%) | .70 | .77 |

**Missing observations: 1 for Race at Wave 1, 1 for Employment at Wave 1, 2 for ADI at both waves, 4 for Alcohol use at Wave 1 and 1 at Wave 3, 1 for CVD at both waves, and 1 for Diabetes at both waves.**

**eTable 5: Sociodemographic differences associated with CVD and diabetes at Wave 3.**

| **Characteristic** | **CVD**  **OR (95% CI)** | **p-value** | **Diabetes**  **OR (95% CI)** | **p-value** |
| --- | --- | --- | --- | --- |
| Age | 1.04 (1.02 to 1.07) | <.001 | 1.00 (1.00 to 1.05) | .03 |
| Race  Black  Native American  White  Other | 2.07 (1.32 to 3.27) 2.17 (0.50 to 9.45) [reference]  0.72 (0.04 to 5.78) | .002  .28  NA  .78 | 1.69 (0.97 to 2.93)  1.76 (0.25 to 8.04) [reference]  1.76 (0.09 to 14.25) | .06  .50  NA  .63 |
| Employment  Unemployed  Retired  Part-time  Other  Full-time | 2.09 (0.08 to 5.59)  1.88 (1.05 to 3.38)  1.38 (0.77 to 2.46)  0.43 (0.16 to 0.98)  [reference] | .13  .03  .28  .06  NA | 2.45 (0.85 to 6.67)  1.09 (0.52 to 2.20)  1.05 (0.50 to 2.11)  0.37 (0.09 to 1.13)  [reference] | .08  .81  .89  .11  NA |
| SES Index | 0.78 (0.67 to 0.91) | .001 | 0.76 (0.63 to 0.91) | .004 |
| ADI | 1.01 (1.00 to 1.03) | .02 | 1.01 (1.00 to 1.03) | .08 |
| Lifetime Smoking History | 1.35 (0.84 to 2.17) | .21 | 0.86 (0.46 to 1.55) | .63 |
| Alcohol Use  Never  Former  Current | [reference]  1.26 (0.66 to 2.39)  0.59 (0.36 to 0.98) | NA  .48  .04 | [reference]  1.27 (0.59 to 2.68)  0.78 (0.42 to 1.45) | NA  .53  .42 |
| BMI* | 1.00 (0.98 to 1.03) | .75 | 1.02 (0.99 to 1.05) | .23 |

SES = socioeconomic status; ADI = area deprivation index; BMI = body mass index

P-values are computed from bivariate logistic regressions comparing the disease group to those without CVD or diabetes

* Note that 3 participants missing BMI at Wave 3 were excluded from this analysis.
